# Association between Malnutrition Assessed by the Global Leadership Initiative on Malnutrition Criteria and Mortality in Older People: A Scoping Review

**DOI:** 10.3390/ijerph20075320

**Published:** 2023-03-30

**Authors:** Dolores Sánchez-Rodríguez, Dorien De Meester, Léa Minon, Marie Claessens, Neslian Gümüs, Siddhartha Lieten, Florence Benoit, Murielle Surquin, Ester Marco

**Affiliations:** 1 Geriatrics Department, Brugmann University Hospital, Université Libre de Bruxelles, Place A. Van Gehuchten 4, 1020 Brussels, Belgium; 2 Rehabilitation Research Group, Hospital del Mar Medical Research Institute (IMIM), Dr. Aiguader, 88, 08003 Barcelona, Spain; 3 Geriatric Department, Centre Fòrum-Hospital del Mar, Parc de Salut Mar, Llull, 410, 08029 Barcelona, Spain; 4 WHO Collaborating Centre for Public Health Aspects of Musculoskeletal Health and Ageing, Division of Public Health, Epidemiology and Health Economics, University of Liège, 4000 Liège, Belgium; 5 Department of Geriatrics, Onze Lieve Vrouw Ziekehuis (OLV) Aalst, Moorselbaan 164, 9300 Aalst, Belgium; 6 Department of Geriatrics, Universitair Ziekenhuis Brussel, Laarbeeklaan 101, Jette, 1090 Brussels, Belgium; 7 Physical Medicine and Rehabilitation Department, Hospital del Mar-Hospital de L’Esperança, Parc de Salut Mar, Pg. Marítim de la Barceloneta, 25-29, 08003 Barcelona, Spain; 8 School of Medicine, Universitat Pompeu Fabra, Plaça de la Mercè, 10-12, 08002 Barcelona, Spain

**Keywords:** GLIM criteria, malnutrition, older people, mortality, muscle mass, comprehensive geriatric assessment

## Abstract

The Global Leadership Initiative on Malnutrition (GLIM) criteria were introduced in 2018 for the diagnosis of malnutrition in adults. This review was aimed at gathering the evidence about the association between malnutrition according to the GLIM criteria and mortality in older people, an emerging and clinically meaningful topic in the implementation of the GLIM criteria in geriatric healthcare settings. This scoping review considered meta-analyses, systematic reviews, cohort studies, and cross-sectional studies published in PubMed, Scopus, and the Cochrane Database for Systematic Reviews from the development of the GLIM criteria in 2018 to January 2023. Seventeen articles (15 cohort and 2 cross-sectional studies) were included. The association between GLIM criteria and mortality had been assessed in hospitalized (11 over the 17 articles) and community-dwelling older populations, and those in nursing homes. The review found a strong association between malnutrition according to GLIM criteria and mortality in hospitalized (1.2-fold to 7-fold higher mortality) and community-dwelling older people (1.6-fold to 4-fold higher mortality). These findings highlight the prognostic value of the GLIM criteria and support strategies towards the implementation of malnutrition evaluation according to the GLIM, in order to optimize comprehensive geriatric assessment and provide older people with the highest quality of nutritional care. Studies in nursing home populations were very scarce and may be urgently required.

## 1. Introduction

In accordance with the World Health Organization (WHO), malnutrition refers to deficiencies or excesses in a person’s intake of nutrients, an imbalance of essential nutrients or impaired nutrient utilization [1,2,3,4]. Malnutrition in adults (or disease-related malnutrition) consists of a combination of weight loss and changes in body composition, compromised food intake or the assimilation of nutrients, and varying degrees of inflammation [1,5]. Malnutrition in adults is associated with adverse functional and clinical outcomes in the general population [6], particularly in the oldest ones. The highest association between malnutrition and adverse outcomes observed in the older population is mostly due to a greater susceptibility to infectious diseases, reduced healing capacity, increased presence of anemia, and higher incidence of sarcopenia and frailty [7,8]. This fact, together with the higher prevalence of malnutrition in older people (ranging from 14.9 to 40.6%) [9], indicates the older population as one of the most vulnerable to malnutrition, but at the same time, indicates that the benefits resulting from nutritional interventions may be greater in older adults [10].

During the past 30 years, many different tools to identify malnutrition have been developed and validated in different populations and settings, constructed based on the key phenotypic and etiologic criteria that characterize individuals with malnutrition, according to the different needs and resources of healthcare systems [5,6,11]. The exact number of these tools is unknown, but an exploratory search found more than 100 options. The large quantity of available tools may mean that a patient could be identified and treated as malnourished or not, depending on the approach used [12].

The societies of clinical nutrition and metabolism have taken action to develop a harmonized framework of operational criteria for the diagnosis of malnutrition in adults, suitable to be applied in all populations, in all medical and surgical specialties, and in all healthcare settings. In 2017, the Global Leadership Initiative on Malnutrition (GLIM) was formed by the European Society of Clinical Nutrition and Metabolism (ESPEN), American Society for Parenteral and Enteral Nutrition (ASPEN), Latin American Federation of Nutritional Therapy, Clinical Nutrition and Metabolism (FELANPE), and Parenteral and Enteral Nutrition Society of Asia (PENSA) [6]. The development of the GLIM criteria was also endorsed by international societies specializing in the ageing process, such as the European Union Geriatric Medicine Society (EuGMS) [6,11].

The GLIM criteria were launched in 2018 and incorporate evidence-based and consensus-based individual criteria that are considered the most important features of malnutrition by the working group. The GLIM criteria consist of a three-step approach: first, screening by any validated tool and second, the diagnosis of malnutrition based on phenotypic and etiologic criteria. Phenotypic criteria are unintentional weight loss, low body mass index (BMI), and reduced muscle mass. Etiologic criteria are reduced food intake or assimilation, or disease burden/inflammation. The diagnosis of malnutrition according to the GLIM requires the presence of at least one etiologic and at least one phenotypic criterion [6]. In a third step, the GLIM recommends grading the severity of malnutrition based on the number of phenotypic criteria fulfilled [5,13]. The presence of the sequential malnutrition risk screening and diagnostic assessment as the first and second phases in the structure of the GLIM criteria is due to the concept of “Nutritional care” and its two sequential steps, i.e., first, the identification of patients’ needs using a validated assessment tool, followed by an in-depth assessment of nutritional status. The clinical and scientific community, supported by the major nutrition societies, are pursuing a clinically relevant diagnosis code for malnutrition in adults according to the GLIM criteria in the next revision of the World Health Organization International Classification of Diseases (ICD-11) [2,5,14].

The guidance on validation of the operational criteria for the diagnosis of protein-energy malnutrition in adults [15] is a consensus document aimed at shedding light on the use of the GLIM criteria in nutritional care, and is meant to be used as a guide for validation studies [15]. It is advised that the GLIM criteria are used as a whole, as each individual criterion is equally important. While most of individual criteria are already part of the standard of care in clinical practice and their use is implemented in clinical settings (both alone or as part of validated questionnaires), the reduced muscle mass criterion is still not universally accepted or implemented in clinical practice [11,16].

The potential inclusion of the GLIM criteria as part of the comprehensive geriatric assessment in clinical healthcare settings requires, first, a demonstration of their capability to improve the knowledge about the prognosis of older people. According to a recent initiative, the Core Outcome Set (COS) for malnutrition intervention studies in older adults [17], 26 different malnutrition-related outcomes have been recently described as the most frequently used ones in intervention studies in this population. Mortality was one of the outcomes found by the COS, a hard, objective, and harmonized outcome, defined in the same terms in every population and setting, independently of the inclusion criteria of the individual studies, which may prevent the potential bias derived from the use of different tools for the outcome measure variable. Moreover, the guidance on validation of the operational criteria for the diagnosis of protein-energy malnutrition in adults points out mortality as one of the relevant, meaningful health outcomes to be used in validation studies for the GLIM criteria [15], and could be helpful to assess the prognosis value for the GLIM criteria in older people.

Since its publication in 2018, the GLIM criteria have aroused the interest of the scientific and clinical community worldwide and many original studies have emerged in different populations, including the oldest ones. To date, one single scoping review has been published to assess the association between malnutrition according to the GLIM criteria and adverse health outcomes [18]. The review was focused on the general adult population aged 18 and over and all types of health outcomes were included. From a total of 79 publications included, 68% of studies included populations other than older adults and 25 (32%) were in the population aged 65 and older; 33 studies (27%) included mortality as an outcome at all ages. The review was not focused on older people and mortality, nor focused on if the GLIM criteria could be helpful as a valuable prognostic tool as part of the comprehensive geriatric assessment of the three main types of geriatric populations and settings. While methodologically robust, the aims and scope of the review were wide and difficult to translate into the daily practice and comprehensive geriatric assessment of older people, because of the wide range of populations and outcomes included [18]. On the other hand, the potential inclusion of the GLIM criteria as part of the comprehensive geriatric assessment requires acknowledgment of their feasibility in clinical practice. For this reason, it would be of interest to explore the methods that have been used so far, particularly for the criterion that has shown to be more challenging, which is the reduced muscle mass phenotypic criterion, in the studies about vital prognosis in all geriatric healthcare settings and older populations. 

Based on these considerations, the primary objective of this review was aimed at gathering evidence in order to assess the association between malnutrition according to the GLIM criteria and all-cause mortality in older people. Secondarily, the evidence about the different assessment methods used for the reduced muscle mass phenotypic criterion in the different populations within the studies which assess mortality was gathered.

## 2. Methods

This is a scoping review, whose structure and procedures were based on the checklist recommended by the Arksey and O’Malley through a six-step process (formulate the question, gather and classify the evidence, critically appraise each article, summarize the evidence, and as final step, write the conclusions) and follows the Preferred Reporting Items for Systematic Reviews and Meta-Analyses extension for Scoping Reviews (PRISMA-ScR) Checklist (Supplementary Material, Table S1) [19].

### 2.1. Population/Concept/Context (PCC)

The PCC of the scoping review are shown in Table 1. 

### 2.2. Information Sources

PubMed, Scopus, and the Cochrane Database for Systematic Reviews were the used resources to carry out the bibliographic search (last search consulted on the 1 January 2023). Authors of unavailable studies were contacted to provide the full-text article when necessary. Additional studies referenced in the selected articles were also consulted (backward citation searching).

### 2.3. Search Strategy

Time and language limits were applied for the search strategy. The bibliographic search was limited to the period from the date where the GLIM criteria were launched in 2018. The final time limit for the bibliographic search was on the 1 January 2023. For the language limit, English was the only selected language for pragmatic reasons. 

The free text terms and vocabulary used for the bibliographic search were as follows: Global Leadership Initiative on Malnutrition (GLIM), mortality, comprehensive geriatric assessment, Geriatric Medicine, older people, older population, geriatrics, malnutrition, muscle mass, muscle strength, community-dwelling, hospitalized people, and nursing home population. Details about the search are provided in the Supplementary Material (Table S1).

### 2.4. Selection Process

Two reviewers (LM and DSR) carried out the bibliographic search. The eligibility criteria were applied in order to select the articles for the review. The procedure to decide which studies were eligible or not for the review was based on discussion between these two reviewers (LM and DSR). When consensus was not achieved, the opinion from a third reviewer (MS) was requested for consensus. The agreement of the two reviewers was calculated as follows: if the agreement was 0, it was considered poor agreement; from 0.0 to 0.2, a slight agreement; from 0.21 to 0.40, fair agreement; from 0.41 to 0.60, moderate agreement; from 0.61 to 0.8, substantial agreement; and from 0.81 to 1, almost perfect agreement [20,21]. The agreement between the two reviewers was 86.6%, which was considered as “almost perfect agreement”.

### 2.5. Data Collection Process

The two reviewers (LM and DSR) who carried out the bibliographic search were also in charge of the data collection process. 

### 2.6. Data Items (Outcomes)

The outcome assessed in the review was all-cause mortality. No specific limit for the follow-up period was applied; consequently, studies of all-time follow-up periods were allowed for the bibliographic search.

### 2.7. Synthetic Method

The process to decide which studies were eligible or not for the review was discussed among the research team. The two reviewers (LM and DSR) in charge of the article selection process synthesized and collected the data from each article. Each selected article was critically read and analyzed, and the relevant information related to the review was included. A summary table was chosen as the method to synthetize and expose the findings of the bibliographic search. A summary table was synthesized for each article and included the following: corresponding author, year of publication, population (hospitalized, community-dwelling, and nursing home older adults), sample size, mean age and standard deviation (SD), primary outcomes, study design, muscle mass assessment methods, and odds ratios (OR) and hazard ratios (HR) with 95% confidence intervals (95%CI). Finally, all manuscripts retrieved were sorted by population. The table was modified in consecutive rounds of consensus among the authors before achieving the final version, which synthesizes the most relevant information of the bibliographic search.

## 3. Results

**Figure 1:**
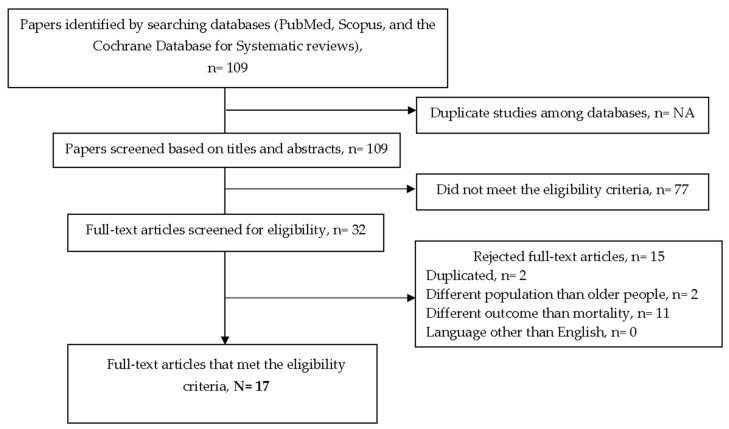
Detailed flow diagram of the bibliographic search.

The search strategy generated 109 references, from which 17 articles were finally included in this review (Figure 1). 

From the 17 articles included, fifteen articles were cohort studies [13,20,22,23,24,25,26,27,28,29,30,31,32,33,34] and 2 were cross-sectional studies [35,36]. No meta-analysis, or systematic reviews, or randomized controlled trials were found. No implementation or feasibility studies were found. Relevant information about the study findings in hospitalized, community-dwelling, and nursing home older populations is provided in Table 2. 

Regarding the study settings and populations, the majority of studies were from the hospital setting (11/17); 11 articles were conducted in the hospitalized older population [22,23,24,29,30,31,32,33,34,35,36], from which 3 were focused on hospitalized older patients with cancer [22,23,24], and 5 studies included community-dwelling older people [13,20,25,27,28]. Only two studies were carried out in nursing home settings [25,26], from which one study included both a community-dwelling and nursing home population [25]. With the exception of one of the studies, which included patients aged 60 ± 12.6 years old [23], the age of the participants in the studies ranged from 65 years old to 84.9 ± 5.3 [31].

Nine of the eleven articles about hospitalized older people were cohort studies [22,23,24,29,30,31,32,33,34] and two were cross-sectional studies [35,36]. The shortest follow-up was 4 months [34] and the longest was 8 years [33]. The smallest sample size was 56 patients [34] and the largest was 6519 patients [36]. The lowest association between patients meeting the GLIM criteria and mortality ranged from OR = 1.231 (95%CI 1.022 to 1.484; *p* = 0.029) [36] to OR = 7.29 (95%CI 1.87 to 28.4; *p* = 0.0043) (i.e., a maximum of a seven-fold higher mortality risk for hospitalized older people who met the GLIM criteria was found) [31]. The age of the participants varied widely in the studies, e.g., in the study with the seven-fold increase [31], the participant population was on average 85 years.

All of the three articles found about hospitalized older people with cancer [22,23,24] were cohort studies, with follow-up ranging from 6 months [23] to 5 years [24]. The smallest sample size was 282 patients [23] and the largest was 1192 patients [22]. The minimal association between patients meeting the GLIM criteria and mortality was an OR = 1.350 (95%CI 1.09 to 1.66; *p* = 0.006) [22], and the maximal association was an OR = 2.72 (95%CI 1.37 to 5.4; *p* = 0.004) (i.e., a maximum of a two-fold to three-fold higher mortality risk for hospitalized older people with cancer who met the GLIM criteria was found) [23]. 

All five articles about community-dwelling populations were cohort studies [13,20,25,27,28]. The follow-up ranged from 2 years [27] to 14 years [28] and the sample size from 534 patients [13,20] to 3702 patients [28]. The weakest association between the GLIM criteria and mortality in patients meeting the GLIM criteria was an OR = 1.62 (95% confidence Interval (CI) 1.39 to 1.89; *p* < 0.01) [28] and the strongest was a HR = 4.41 (95%CI 2.17 to 8.97) (i.e., a maximum of a four-fold higher mortality risk for community-dwelling older people who met the GLIM criteria was found) [13].

Both of the articles conducted in nursing home settings were cohort studies [25,26], with 1 year [26] and 2 years of follow-up [25] and a sample size of 485 patients [26] and 2032 patients [25], respectively. The minimal association between patients meeting the GLIM criteria and mortality was a HR = 1.37 (95%CI 1.12 to 1.66; *p* = 0.002) [25], and the maximum association was a HR = 2.41 (95%CI 1.36 to 4.27; *p* < 0.01) (i.e., a maximum of a two-fold higher mortality risk for nursing home population who met the GLIM criteria was found) [26].

The assessment method for reduced muscle mass as the phenotypic criterion varied widely: five studies used dual energy X-ray absorptiometry (DXA) [13,20,23,28,34], five studies used bioimpedance analysis (BIA) [23,29,30,31,33], five studies used handgrip strength as a surrogate marker of muscle mass [20,23,26,27,32], nine studies used calf circumference, mid-upper arm circumference, and/or other anthropometric measures [20,22,23,25,26,32,33,34,36], two studies used computed tomography scan (CT) [23,24], and none used magnetic resonance imaging or muscle ultrasound. 

## 4. Discussion

This scoping review shows a strong association between malnutrition according to the GLIM criteria and all-cause mortality in hospitalized, community-dwelling, and nursing home older populations. This association ranged from a 1.2-fold [36] to 7-fold [31] higher mortality in hospitalized populations [31], from a 1.6-fold [28] to 4-fold higher mortality in community-dwelling populations [13], and there was a 2-fold higher mortality in nursing home populations [26]. No implementation or feasibility studies have been found and the studies in nursing home settings were very scarce. 

To the authors’ knowledge, this is one of the very few literature syntheses about malnutrition according to the GLIM criteria in older people. The previous scoping review by Correia et al. [18] had much broader objectives, which were “how the GLIM criteria have been used in published literature and compare the reported validation methods to published validation guidance”, which is different from the focused objective about a clinically meaningful outcome (prognosis) in our study. The population also differs between the two, as the previous review included a general adult population aged 18 and over; 68% of studies included populations other than older adults (e.g., general population, patients with cancer, patients with COVID-19, and with gastrointestinal, renal, and cardiovascular diseases). From a total of 79 publications included, only 13 studies were about older people and mortality [18]. This is consistent with the results of our review (17 studies), which include those studies that have been published after Correia et al., completed their search. The findings by Correia et al. are remarkable and the methodological quality of the manuscript is very robust, but did not answer the research question of our study, which was to explore the ability of the GLIM criteria to predict death in all-type of geriatric settings and populations. The findings of the review are innovative, clinically meaningful, and support potential strategies towards the inclusion of the GLIM framework as part of the comprehensive geriatric assessment in geriatric populations and settings. 

The selection of mortality as outcome in the review was due to three reasons: First, because death is a hard outcome, objective to measure, and defined in the same terms in the individual studies, so the decision was aimed at decreasing biases and helping the study findings to be easily interpreted by researchers, clinical geriatricians and gerontologists, who are, in the end, the intended end-users of the GLIM criteria. The second reason was a pragmatic one, because a preliminary search had been conducted and shown a relatively limited number of studies in older people, where mortality was highlighted as the most frequent outcome used in the individual studies. Finally, because death had been identified as a meaningful health outcome by the COS for malnutrition intervention studies in older adults [17] and in the guidance on the validation of the operational criteria for the diagnosis of protein-energy malnutrition in adults [15]. 

The prognosis value of a reduced muscle mass determined its inclusion as one of the GLIM phenotypic criteria [16,37,38]. In the review, the most frequently used methods to assess muscle mass were DXA (three studies of the five conducted in community-dwelling population) [13,20,28] and anthropometric measures (8 studies of the 11 in hospitalized population) [22,23,25,26,32,33,34,35]. These choices in the assessment technique in each study might be due to the characteristics of the technique itself, but also to the setting and population. Five studies in our review used muscle strength as a surrogate marker of muscle mass [20,23,26,27,32]. In order to face the challenge of assessing muscle mass in different settings and populations, and aim at facilitating the widest use of the GLIM criteria, the ESPEN recently launched the “Guidance for assessment of the muscle mass phenotypic criterion for the GLIM diagnosis of malnutrition” in 2022, where several techniques, approaches, and their correspondent thresholds for the assessment of muscle mass or its surrogate markers were recommended for research and clinical practice [16]. In this respect, it is worth mentioning that the ESPEN guidance recommends not to assess muscle strength as a surrogate marker of muscle mass [16]. 

This review did not find any studies where the muscle mass criterion was assessed by ultrasound. This technique has been recommended not only by the ESPEN guidance, but it has been also pointed out by the EuGMS as a feasible, inexpensive, and innocuous technique for the assessment of muscle mass [39], as well as a promising technique with potential to be included as part of the GLIM criteria [16] and the comprehensive geriatric assessment [37,39]. Studies assessing muscle mass measured by ultrasound are urgently needed. 

Several limitations and strengths of this scoping review should be acknowledged. The first limitation is the non-systematic nature of the review, which might not be a major issue to provide an updated overview, especially given the relatively limited number of original studies. According to Grant and Booth, scoping reviews are a preliminary assessment of potential size and scope of research literature [40]. Scoping reviews are best designed when a body of literature has not yet been comprehensively reviewed or exhibits a complex or heterogeneous nature not amenable to a more precise review; they are also useful to identify gaps in existing research [41]. To the authors’ knowledge, the findings are novel, the review is valuable, and conclusions are sound, consistent, and clinically meaningful. Second, the review included those studies that assess malnutrition according to the GLIM criteria, which may involve a selection bias related to the choice of the assessment tool itself and the accessibility of the settings and populations to the techniques required for muscle mass assessment. It is worth emphasizing that the majority of studies were from hospital settings (11/17) and that, despite some studies were conducted in community-dwelling older people, due to the large diversity and wide range of characteristics of this particular population, this group also warrant further research. This source of bias may impact the generalizability of the findings to those settings where there are fewer studies and the access to techniques may be more challenging, such as nursing homes. This may be one of the reasons to explain why the studies in the nursing home population were so scarce, which may limit the generalization of the findings within this population and setting, and further studies in this healthcare setting are urgently required. The high association between malnutrition and mortality observed in hospitalized older people may be biased due to the influence that malnutrition itself may cause in the hospitalizations, which was an outcome not in the scope of this review. Third, malnutrition is a complex condition involving a large quantity of potential adverse outcomes for patient health, and the review is only focused on all-cause mortality. Further studies are required to assess the association between the GLIM criteria and outcomes other than mortality, such as hospital (re)admissions, diagnostic performance indicators, feasibility of the implementation in clinical practice, especially in older adults, where patient-centered outcome measures such as quality of life and daily functioning are crucial. Finally, the study was not designed to provide an exhaustive overview of the muscle mass assessment method used in all studies about GLIM criteria in older people, but only in those that include mortality as outcome. The findings of this review support the updated recommendations of the clinical nutrition and geriatric scientific societies that seek to provide the highest quality of nutritional care in older people.

## 5. Conclusions

This scoping review gathered evidence about the association between malnutrition according to the GLIM criteria and mortality in the older population. The review showed that the association between GLIM criteria and mortality had been assessed in hospitalized (11 over the 17 articles), community-dwelling older populations and those in nursing homes. The review found a strong association between malnutrition according to GLIM criteria and mortality in hospitalized (1.2-fold to 7-fold higher mortality) and community-dwelling older people (1.6-fold to 4-fold higher mortality). The majority of studies were from hospital settings (11/17) and, despite some studies being conducted in community-dwelling older people, due to the large diversity and wide range of characteristics of this particular population, this group also warrant further research. Studies in the nursing-home population were very scarce and may be urgently required. Studies assessing the challenge of treating malnutrition according to the GLIM criteria or feasibility studies were not found and they may also be required. The most frequent methods to assess muscle mass as a phenotypic criterion were DXA and anthropometric measures. Further research is needed regarding the evaluation of the GLIM criteria compared with other tools, in terms of mortality prediction. These findings highlight the prognostic value of the GLIM criteria and support strategies towards the implementation of malnutrition evaluation according to the GLIM, in order to optimize comprehensive geriatric assessment and provide older people the highest quality of nutritional care.

## Figures and Tables

**Table 1:** Inclusion criteria of the scoping review (PCC).

**Population**	Human patient population. Age ≥ 65-year-old. Any condition or disease state. Any healthcare geriatric setting (hospitalized, community-dwelling, and nursing home population).
**Concept**	Articles about the GLIM criteria Articles published from 3 September 2018 (date when the GLIM criteria were launched) to 1 January 2023 (date when the last bibliographic search for the review was consulted). All-cause mortality as primary or secondary outcome.
**Context**	Full-text, peer-reviewed publications in indexed journals. All type of study designs were included in the bibliographic search. Utilization of the GLIM criteria. Written in English.

**Table 2:** Summary of findings from the bibliographic search (from 1 September 2018 to 1 January 2023) of the scoping review, ordered according to the three populations assessed (hospitalized, community-dwelling, and nursing home older populations) (n = 17).

First Author, Year of Publication, and Citation	Population	Sample Size (n)	Mean Age (Years) ± SD	Primary Outcome: Mortality. Other Secondary Outcomes	Design	Muscle Mass Assessment Method	Odds Ratio/Hazard Ratio (95%CI; *p*-Value)
Hirose, 2021 [29]	Hospitalized patients	1332	Aged 65 and older	Mortality	Cohort study	BIA	HR = 1.57 (95%CI 1.09–2.27; *p* = 0.016)
Gomes Pereira, 2021 [35]	Hospitalized patients	90	68.0 (56.3–5.3)	Mortality	Cross-sectional study	Omission of muscle mass as phenotypic criterion	OR = 1.498 (95%CI 0.496–4.521; *p* = 0.473)
Davalos-Yerovi, 2021 [30]	Hospitalized patients with stable COPD	200	66.5 ± 9	Mortality Hospitalization, length of stay	Cohort study	BIA	HR = 2.8 (95%CI 0.9–8; *p* = 0.005)
Allepaerts, 2020 [31]	Hospitalized patients	79	84.9 ± 5.3	Mortality Institutionalization	Cohort study	BIA	OR = 7.29 (95%CI 1.87–28.4; *p* = 0.0043)
Xu, 2020 [36]	Hospitalized patients	6519	78.4 ± 6	Mortality Diagnostic performance indicators of several cut-off points of calf circumference	Cross-sectional study	Calf circumference	OR = 1.231 (95%CI 1.022–1.484; *p* = 0.029)
Munoz, 2021 [32]	Hospitalized patients in emergency wards	165	73 (65–102)	Mortality Length of stay, transfer to intensive unit care, and the diagnostic performance indicators	Cohort study	Handgrip strength, calf circumference, subscapular skinfold thickness, triceps skinfold thickness, adductor pollicis thickness.	HR = 4.23 (95%CI 1.2–14.9; *p* = 0.02)
Sanz-Paris, 2020 [33]	Hospitalized patients with type 2 diabetes	159	77.9	Mortality Frailty	Cohort study	Calf and mid-arm circumference	HR = 2.09 (95%CI 1.29–3.38; *p* = 0.003)
Sobestiansky, 2021 [34]	Hospitalized	56	84 ± 7.3	Mortality Malnutrition and sarcopenia	Cohort study	DXA and calf circumference	HR = 4.83 (95%CI 1.04–22.39)
Zhang, 2021 [22]	Hospitalized patients with cancer	1192	Aged 65 and older	Mortality	Cohort study	Calf circumference	HR = 1.35 (95%CI 1.09–1.66; *p* = 0.006)
Contreras-Bolívar, 2019 [23]	Hospitalized patients with cancer	282	60 ± 12.6	Mortality	Cohort study	DXA, BIA, CT, handgrip strength, mid-arm circumference, and arm muscular circumference	If using handgrip strength, OR = 2.72 (95%CI 1.37–5.4; *p* = 0.004) If using FFMI, OR = 1.87 (95%CI 1.01–3.48; *p* = 0.047)
Huang, 2021 [24]	Hospitalized patient with cancer	597	72 ± 8	Mortality	Cohort study	CT	OR = 1.360 (95%CI 0.942–1.963; *p* = 0.101)
Rodríguez-Mañas, 2021 [27]	Community-dwelling	1294	75 ± 6.29	Mortality Incident frailty	Cohort study	Handgrip strength	OR = 1.758 (95%CI 1.073–2.849; *p* < 0.05
Sanchez-Rodriguez, 2021 [20]	Community-dwelling	534	73.07 ± 5.96	Mortality	Cohort study	DXA, handgrip strength, calf circumference, mid-arm circumference, Goodman grid, Ishii score chart, Yu formula, and omission of muscle mass as phenotypic criterion	HR = 3.38 (95%CI 1.89–6.09)
Sanchez-Rodriguez, 2020 [13]	Community-dwelling	534	73.2 ± 6.05	Mortality Falls, fractures, and institutionalization	Cohort study	DXA	HR = 4.41 (95%CI 2.17–8.97)
Yeung, 2021 [28]	Community dwelling	3702	72 ± 4	Mortality Onset of sarcopenia, frailty, falls, mobility limitation, and hospitalization	Cohort study	DXA	HR = 1.62 (95%CI 1.39–1.89; *p* < 0.01)
Yeung, 2021 [25]	Community-dwelling and institutionalized patients	2032	CD: 78.1 ± 6.5 I: 85.5 ± 6.4	Mortality	Cohort study	Corrected muscle mass arm circumference	HR = 1.37 (95%CI 1.12–1.66; *p* = 0.002)
Sanz-Paris, 2021 [26]	Institutionalized patients	485	84.71	Mortality	Cohort study	BIA, handgrip strength, and calf circumference	Min: HR = 0.81 (95%CI 0.46–1.42; *p* = 0.0456) Max: HR = 2.41 (95%CI 1.36–4.27; *p* < 0.01)

BIA: Bioelectrical impedance analysis; CI: confidence interval; CT: computed tomography; DXA: dual energy X-ray absorptiometry; FFMI: fat-free mass index; GLIM: Global Leadership Initiative on Malnutrition; HR: hazard ratio; MNA-SF: mini-nutritional assessment-short form; MR: magnetic resonance; NA: not applicable; OR: odds ratio. From the 17 articles found in the review, 11 articles were about the hospital setting, 5 about a community-dwelling population, and 2 were about a nursing home setting, from which 1 article included both a community-dwelling and nursing home population.

## Data Availability

Not applicable.

## Supplementary Materials

The following supporting information can be downloaded at: https://www.mdpi.com/article/10.3390/ijerph20075320/s1, Table S1: Preferred Reporting Items for Systematic reviews and Meta-Analyses extension for Scoping Reviews (PRISMA-ScR) Checklist, and Table S2: Search strategy.

## Abbreviations

ASPEN: American Society for Parenteral and Enteral Nutrition; ASN: American Society for Nutrition; BIA: Bioelectrical impedance analysis; BMI: Body mass index; CI: Confidence Interval; CT: Computed tomography; DXA: Dual energy x-ray absorptiometry: European Society of Clinical Nutrition and Metabolism; EuGMS: European Union Geriatric Medicine Society; GLIM: Global Leadership Initiative on Malnutrition; HR: Hazzard ratio; FELANPE: Federacion Latino Americana de Terapia Nutricional, Nutricion Clinica y Metabolismo; MNA-SF: Mini-nutritional assessment-short form; MR: Magnetic resonance; NA: Not applicable; OR: Odds ratio; PENSA: Parenteral and Enteral Nutrition Society of Asia; PRISMA-ScR: Preferred Reporting Items for Systematic reviews and Meta-Analyses extension for Scoping Reviews; SD: Standard deviation.
