# Supplementary material for: Association between Malnutrition Assessed by the Global Leadership Initiative on Malnutrition Criteria and Mortality in Older People: A Scoping Review
Source: Int J Environ Res Public Health. 2023 Mar 30;20(7):5320. doi: 10.3390/ijerph20075320 (PMC10094645; doi:10.3390/ijerph20075320)
Supplement: Supplementary file 1 [file ijerph-20-05320-s001.zip › Table S2.pdf]

Table S2. Search strategy.

Pubmed

Search: **((glim criteria) AND (older people)) OR ((glim criteria) AND (mortality))**

("glim"[All Fields] AND ("criteria s"[All Fields] OR "criterias"[All Fields] OR "standards"[MeSH Subheading] OR "standards"[All Fields] OR "criteria"[All Fields]) AND (("older"[All Fields] OR "olders"[All Fields]) AND ("people s"[All Fields] OR "peopled"[All Fields] OR "peopling"[All Fields] OR "persons"[MeSH Terms] OR "persons"[All Fields] OR "people"[All Fields] OR "peoples"[All Fields]))) OR ("glim"[All Fields] AND ("criteria s"[All Fields] OR "criterias"[All Fields] OR "standards"[MeSH Subheading] OR "standards"[All Fields] OR "criteria"[All Fields]) AND ("mortality"[MeSH Terms] OR "mortality"[All Fields] OR "mortalities"[All Fields] OR "mortality"[MeSH Subheading]))

Translations

criteria: "criteria's"[All Fields] OR "criterias"[All Fields] OR "standards"[Subheading] OR "standards"[All Fields] OR "criteria"[All Fields]

older: "older"[All Fields] OR "olders"[All Fields]

people: "people's"[All Fields] OR "peopled"[All Fields] OR "peopling"[All Fields] OR "persons"[MeSH Terms] OR "persons"[All Fields] OR "people"[All Fields] OR "peoples"[All Fields]

criteria: "criteria's"[All Fields] OR "criterias"[All Fields] OR "standards"[Subheading] OR "standards"[All Fields] OR "criteria"[All Fields]

mortality: "mortality"[MeSH Terms] OR "mortality"[All Fields] OR "mortalities"[All Fields] OR "mortality"[Subheading]

Scopus

Cochrane Database for Systematic Reviews (CDSR)
